# Effect on Osteoclast Differentiation and ER Stress Downregulation by Amygdalin and RANKL Binding Interaction

**DOI:** 10.3390/biom12020256

**Published:** 2022-02-04

**Authors:** Nguyen Minh Trang, Eun-Nam Kim, Hyun-Su Lee, Gil-Saeng Jeong

**Affiliations:** 1 College of Pharmacy, Keimyung University, Daegu 42601, Korea; ngminhtrang52@gmail.com (N.M.T.); enkimpharm@gmail.com (E.-N.K.); 2 School of Medicine, Kyungpook National University, Daegu 41566, Korea; hyunsu.lee@knu.ac.kr; 3 College of Pharmacy, Chungnam National University, Daejeon 34134, Korea

**Keywords:** amygdalin, RANKL, osteoclast, ROS, ER stress, molecular docking

## Abstract

Bone diseases such as osteoporosis are the result of osteoclast over-activation. There are many therapeutic agents from natural compounds inhibiting the formation of osteoclast that have been reported and are continuously being interested. Amygdalin (AD) is isolated from seeds of *Prunus armeniaca* L. which has many pharmaceutical effects; however, the effect of AD on osteoclast formation and function remains unknown. Therefore, the underlying mechanism of AD on RANKL-induced osteoclast in RAW 264.7 cells was investigated. Molecular docking simulation revealed that AD can bind to the active sites of RANKL with negative binding affinities. Through TRAP activity, bone resorption, and migration, AD effectively inhibited osteoclast differentiation and function. Expression of transcription factors, such as NFATc1, c-fos, and osteospecific genes (including *dcstamp*, *acp5*, *ATP6v0d2*, and *ctsk* results) showed an osteoclast differentiated inhibitory effect by AD treatment. In addition, RANKL-induced activation of MAPK, ER stress, and ROS levels in RANKL-induced osteoclast was significantly inhibited while antioxidant enzymes were recovered in the presence of AD. These results suggest that AD may be a potential candidate derived from natural sources for the treatment of osteoclast bone-related diseases.

## 1. Introduction

Bone, which is the firm organ of the body, establishes the skeleton and supports the musculoskeletal system [1]. The resorption and formation of bone, which includes osteoclasts and osteoblasts, respectively, are stable for the maintenance of normal skeletal structure, function, and mineral homeostasis. Once the state was disturbed, several bone-related diseases will occur [2,3]. The osteoclast is a multinucleated cell that originated from the hematopoietic progenitor of the monocyte–macrophage lineage, which fuse together to become tartrate-resistant acid phosphatase (TRAP)-positive multinucleated cells that act on the bone resorption while osteoblast participates in bone formation and mineralization, which forms a protective layer over the surface of bone [1]. 

Reactive oxidant species (ROS), which are known for their cytotoxicity and damage, play a crucial role in regulating the molecular signals of osteoclast differentiation and ROS activate osteoclast differentiation through the RANKL signaling pathway [4]. The endoplasmic reticulum (ER) is a critical organelle in eukaryotic cells responsible for protein folding, maturation, quality control, and trafficking. ER stress is the state of the protein folding process interruption and unfolded or misfolded protein accumulation by pathologic stimuli responses including oxidative stress, and ROS can regulate the function of ER [5,6]. It is reported that during RANKL-induced osteoclastogenesis, the production of intracellular ROS is stimulated by a signal cascade, ROS has a function as an intracellular signaling medium for cell differentiation into osteoclasts, and ER stress results from oxidative stress caused by ROS during osteoclastogenesis [6]. Besides, inflammation has an important role in skewing bone remodeling toward resorption through the interaction of inflammatory mediators and their related peptides with osteoblasts, osteoclasts, and other immune cells to change the expression of RANK and RANKL, which leads to several bone diseases, such as alveolar bone erosion, periodontitis, rheumatoid arthritis, and septic prosthetic loosening [7].

Molecular docking is an effective computational method for in silico screening, important for the development of drug design [8,9]. Based on the energy and geometrics, docking virtually predicts the binding between two or more binding partners as biological macromolecules (such as protein) or small molecules (such as ligands and drugs) [8,9]. For non-covalent binders, different types of protein–ligand interactions include ionic interactions, hydrogen bonds, and van der Waals interactions in which dispersion, polar, and induced interactions are involved [9]. Among many systemic hormones and cytokines involved in osteoclast differentiation and function, the receptor activator of nuclear factor (NF)-κB ligands (RANKL) is a key osteoclastogenic molecule [10,11]. Previous research reported that baicalin specifically binds to the active site of RANKL and inhibits osteoporosis by regulation of the RANK/RANKL/OPG pathway in vitro and in vivo through bone modeling transcription factors, such as nuclear factor κB2 (nfκb2), TNF receptor-associated factor 6 (traf6), forkhead box O1 a2 (foxo1a2), mitogen-activated protein kinase 8b (mapk8b), TNF superfamily member 13b (tnfsl3b), and bone tissue mineralization, growth, and development in the dexamethasone (DEX)-induced osteoporosis zebrafish larvae model [12]. Therefore, it is important to understand the interaction between RANKL and secondary compounds for further treatment of osteoclast differentiation and bone diseases. 

Natural products play an important role in the development for the treatment of many diseases, and several plant extracts and metabolites exhibit pharmaceutical activities [13]. *Prunus armeniaca* L., a fragrant and flavorful apricot belonging to the Rosaceae family, has its origins in Central Asia and is widely distributed around the world [14,15]. According to published reports, many secondary metabolites, such as steroids, norisoprenoids, terpenoids, proanthocyanidins, and phenolic compounds, have been isolated and structurally elucidated in many parts of *P. armeniaca* [16,17]. Amygdalin (AD), a cyanogenic glycoside isolated from *P. armeniaca*, was reported to exhibit various pharmacological effects, such as anti-fibrosis, anti-inflammation, analgesia, auxiliary anticancer, immunoregulation, anti-atherosclerosis, anti-cardiac hypertrophy, anti-ulcer, hypoglycemia effects, anticholinesterase, and neuroprotective activities [18,19,20,21]. It is reported that the high concentration of AD (10 mg/mL) reduced the viability and size of osteoblasts, decreased extracellular matrix mineralization by down-regulating the COL1A1 and ALPL genes, and enhanced osteoclast formation and bone resorption by up-regulating the TNFSF11 and WNT5A genes while increasing the expression of BGLAP gene. In lower concentrations (0.1 mg/mL), there is almost no effect on functions of human osteoblasts in vitro [22]. A recent study reported that by using MSC-specific transforming growth factor- (TGF-) β receptor 2 conditional knockout (KO) mice (Tgfbr2Gli1-Cre) and C3H10 T1/2 murine mesenchymal progenitor cells, the migration and differentiation of mesenchymal stem cells (MSCs) accelerated the fracture healing process by AD through the TGF-β/Smad signaling pathway, supporting the use of amygdalin-based therapy for fracture healing [23]. However, as reported, there is no information about the effect of AD on osteoclast differentiation and function.

In this study, we explored the binding of AD and RANKL through molecular docking simulation and investigated the effects of AD on osteoclast differentiation and function through the RANKL-induced RAW264.7 in vitro model.

## 2. Materials and Methods

### 2.1. Reagents and Chemicals

Recombinant soluble RANKL ligand (sRANKL) was acquired from PeproTech EC Ltd. (London, UK). An Acid Phosphatase Assay Kit, 3-(4,5-Dimethylthiazol-2-yl)-2,5-diphenyltetrazolium bromide (MTT), leukocyte acid phosphatase, 4-6-diamidino-2-phenylindole (DAPI), ascorbic acid, and β-glycerophosphate were purchased from Sigma-Aldrich Fine Chemicals (Saint Louis, MO, USA). Dulbecco’s modified Eagle’s medium (DMEM), minimum essential medium alpha (α-MEM), and fetal bovine serum (FBS) were purchased from Welgene Bioscience (Daegu, Korea). TRIZOL reagent was purchased from JBI (Seoul, Korea) and TOPscript™ RT DryMIX (dT18 plus) was purchased from Enzynomics (Daejeon, Korea). TB Green^®^ Pre-mix Ex Taq™ II (Tli RNaseH Plus) was purchased from Takara (Tokyo, Japan). Primary antibodies for phospho-JNK, JNK, phospho-p38, p38, phospho-ERK, ERK, BIP, p-eIF2α, eIF2α, and rabbit polyclonal antibodies were purchased from Cell Signaling Technology Inc. (Danvers, MA, USA). Catalase (CAT) and superoxide dismutase (SOD) antibodies were acquired from BD biosciences (San Jose, CA, USA). An anti-nuclear factor of activated T cells-c1 (NFATc1) antibody was acquired from BD biosciences (San Jose, CA, USA). The enhanced chemiluminescence (ECL) Western blotting detection system was purchased from Advansta Inc. (San Jose, CA, USA). 

### 2.2. Isolation and Identification of Amygdalin from the Seeds of Prunus armeniaca L.

The dried seeds of *P. armeniaca* L. (1.0 kg) were extracted three times with EtOH (1 L × 3 h) under reflux. After concentrating under reduced pressure, the EtOH extract (89.7 g) was suspended in distilled water and partitioned with n-hexane, CH_2_Cl_2_, EtOAc, and n-butanol to yield an n-hexane extract (50 g), a CH_2_Cl_2_ extract (8.8 g), an EtOAc extract (9.6 g), an n-butanol extract (6.5 g), and a water layer. The n-butanol extract was chromatographed with column chromatography (CC) on silica gel using stepwise eluent of CH_2_Cl_2_–acetone (gradient 10:1–1:1, *v*/*v*) and then CH_2_Cl_2_–MeOH–H_2_O (gradient 6:1:0.1–1:1:0, *v*/*v*) to obtain 6 fractions (A–F). Compound 1 (35 mg) was isolated from fraction F (177.5 mg) by reserved phase C18 (RP-18) CC using MeOH–H_2_O (3:1, *v*/*v*) as an eluent, followed by preparative high-performance liquid chromatography (HPLC) with an isocratic mixture solvent, 60% MeOH in H_2_O, at 5 mL/min for 60 min. The nuclear magnetic resonance (NMR) spectra of compound 1 (^1^H and ^13^C) were conducted using a 500 MHz NMR spectrometer (JEOL, JNM-ECA 500) with tetramethylsilane (TMS) as an internal standard. NMR solvent DMSO-*d*_6_ was purchased from Sigma-Aldrich (MO, USA). The structure of compound 1 was confirmed as amygdalin using spectroscopic analysis of the NMR data and by comparison with other studies [24,25] (Table S1, Figures S1 and S2).

### 2.3. Molecular Docking Simulation

The crystal structure of the extracellular domain of mouse RANK ligand with the resolution of 2.20 Å was downloaded from the RSCB Protein Data Bank website (PDB ID: 1IQA) [26]. The crystal structure was prepared for docking using Discovery Studio v21.1 (Accelrys, Inc., San Diego, CA, USA). The binding of RANKL and water molecules were removed from the structure for docking simulation using AutoDockTools (ADT). AutoDock Vina 1.1.2 was utilized to perform molecular docking to understand the interaction and binding of AD with RANKL. For the docking procedure, the 3D structure of AD was constructed using ChemDraw 20.1 and Chem3D 20.1 (PerkinElmer, INC., MA, USA) for 2D and 3D conformations, respectively. Automated docking simulation was performed using ADT to assess the appropriate binding orientations and conformations of the ligand molecules with the protein inhibitor. The Lamarckian genetic algorithm (LGA) method implemented in AutoDock Vina was employed. For docking calculations, Gasteiger charges were added by default, the rotatable bonds were set by the AutoDock tools, and all torsions were allowed to rotate. The binding effect of RANKL residues, following the binding affinity score and the root-mean-square deviation (RMSD), was considered as the best molecular interaction. Discovery Studio v21.1 was used for graphic display and analysis of the interactions between AD and the target protein. Various interactions, such as hydrogen bonds, van der Waals interactions, π–π bonds, and the interaction distance between amino acids and the active sites of compounds, were plotted. Van der Waals interactions were detected between AD and the bonding point by hydrophilic and hydrophobic contact.

### 2.4. Cell Culture and Osteoclast Differentiation of RAW 264.7 Cells

RAW 264.7 murine macrophage cells were acquired from the American Type Culture Collection (ATCC, Rockville, MD, USA). RAW 264.7 cells were seeded at 5 × 10^3^ cells/well in 24-well plates and grown in Dulbecco’s modified Eagle’s medium (DMEM) high glucose supplemented with 10% fetal bovine serum (FBS), 100 U/mL of penicillin, and streptomycin. After then, RAW 264.7 cells were incubated for 24 h at 37°C in a humidified atmosphere containing 5% CO_2_. Then, each well was changed to minimum essential medium alpha (α-MEM) containing 10% FBS, 100U/mL penicillin, and streptomycin. After being treated or untreated with RANKL (50 ng/mL), cells were processed with AD at different concentrations (5, 10, 20, 40 µM) for 5 days. The medium was refreshed every day. Osteoclast differentiation was identified by TRAP staining, activity, and other experimental methods.

### 2.5. Cell Viability

The cytotoxic effect of AD on RAW 264.7 cells was detected by an MTT assay according to the following procedure. RAW 264.7 (5 × 10^3^ cells/well) cells were seeded in 96-well plates and maintained at 37 °C in a humidified 5% CO_2_ incubator for 24 h. Cells were treated in the presence or absence of different concentrations of AD for 5 days. Then, 4,5-dimethylthiazol-2-thiazolyl)-5-diphenyltetrazolium bromide (MTT) (5 mg/mL) was added to each well. Next, cells were incubated for 4 h before the supernatant was replaced with dimethyl sulfoxide (DMSO) to dissolve formazan crystals. Cell viability was exhibited at the 540 nm absorbance in a microplate reader (TECAN infinity pro 2000, Männedorf, Switzerland). The results shown are representative of three independent experiments. After trypan blue staining was also treated in the presence or absence of different concentrations of AD for 5 days, RAW 264.7 cells were stained with 0.4% trypan blue and photographed under an optical microscope (CKX53, Olympus, Tokyo, Japan). The amount of positive cells was estimated by counting stained and unstained cells. 

### 2.6. Cell Migration

RAW 264.7 cells were seeded at the density of 1 × 10^3^ cells/well in 6-well plates. Then, cells were incubated for 24 h followed by a constant scratching before being treated or untreated with RANKL (50 ng/mL) and the indicated AD concentration. The amount of migration of yellow marked cells in the indicated red line was measured on the first day and fifth day after treatment using Incucyte^®^ Live Cell analysis systems. The only RANKL-treated group was used as a normalized percentage.

### 2.7. TRAP Staining and Activity

RAW 264.7 cells (1 × 10^3^ cells/well) were cultured in 24-well culture plates in DMEM containing 10% FBS, 100 U/mL of penicillin, and streptomycin. After being incubated for 24 h, the medium was changed to α-MEM containing RANKL (50 ng/mL), and cells were treated with AD at different concentrations. The medium was refreshed every day. After 5 days of incubation, the medium was removed and washed twice with phosphate-buffered saline (PBS). Then, cells were fixed with 4% formaldehyde for 15 min and washed with PBS. Osteoclast differentiation was measured by the Acid Phosphatase Assay Lit (Cat. No. CS 0740) and acid phosphatase leukocyte (Cat. No. 387A-1KT) from Sigma-Aldrich Fine Chemicals (Saint Louis, MO, USA) according to the instruction of the manufacturer. Briefly, fixed cells were reacted with a leukocyte acid phosphatase kit at 37 °C, 5% CO_2_ for 1 h, and then the reaction was conducted in a shaded state. Next, cells were washed three times with PBS and TRAP-positive multinuclear cells, which have three or more nuclei, according to optical microscopic observations.

### 2.8. Actin Ring and DAPI Staining

Alexa 488 Phalloidin (Invitrogen, Carlsbad, CA, USA) was used to measure the degree of differentiation of osteoclasts by staining the ring part. RAW 264.7 cells were maintained in the medium including processed or unprocessed AD for 5 days. Cells were fixed in 4% formaldehyde for 15 min followed by a treatment with 0.5% Triton X-100 solution to permeabilize the cells. Afterward, the experiment was conducted in the dark by staining cells with Alexa 488 for 1 h, then contrasted staining for nuclei with DAPI for 30 min followed by cold PBS washing. Actin rings and DAPI staining in mature osteoclasts were observed with a fluorescence microscope (Nikon Co., Tokyo, Japan). 

### 2.9. Bone Resorption Assay

RAW 264.7 cells (1 × 10^3^ cells/well) were cultured on a Corning osteo assay surface well (Corning, NY, USA). After 24 h of incubation, cells were refreshed with α-MEM medium containing 5% FBS and treated or untreated with RANKL (50 ng/mL) and the indicated AD. The cells were maintained for 7 days at 37 °C in a humidified incubator containing 5% CO_2_. The medium was changed every day. Subsequently, the medium was removed, cells were washed twice with PBS. Cells were detached by 5% sodium hypochlorite for 5 min, washed with PBS, and dried. The surface of each well was visualized using a microscope (Olympus, Tokyo, Japan) and measured with an ImageJ program. 

### 2.10. Measurement of Intracellular Reactive Oxygen Species

RAW 264.7 (5 × 10^3^ cells/well) was dispended in 24-well plates for 24 h. After treatment with RANKL (50 ng/mL), cells were treated with AD at different concentrations and maintained with α-MEM supplemented with 10% FBS, 100U/mL penicillin and streptomycin for 24 h. 2′, 7′-dichlorodihydrofluorescein diacetate (DCF-DA) was utilized to study the production of ROS. After incubation for 20 min at 37 °C in the dark, cells were washed with PBS and fixed with 4% paraformaldehyde (pH 7.4) for 20 min. ROS was detected with a fluorescent Olympus IX microscope 71-F3 2PH (Tokyo, Japan). 

### 2.11. Measurement of Antioxidant Enzyme Activity

Antioxidant enzymes were measured using a Superoxide Dismutase Assay Kit (Cayman chemical, Ann arbor, MI, USA) and a Catalase Assay Kit (Cayman chemical, Ann arbor, MI, USA), respectively. RAW 264.7 cells treated with AD and RANKL were recovered using extraction buffer (20 mM HEPES buffer, pH 7.2, 1 mM EGTA, 210 mM mannitol, 70 mM sucrose) and centrifuged, while SOD was measured in a 450 nm ELISA plate reader from the recovered supernatant. Then, CAT was measured at 540 nm with an ELISA plate reader from the supernatant recovered using the extraction buffer (50 mM potassium phosphate, pH 7.0, 1 mM EDTA) according to the manufacturer’s instructions.

### 2.12. Real-Time Quantitative PCR

Real-time PCR was used to detect the expression of *dcstamp*, *acp5*, *atp6d0v2*, and *ctsk*. The cell lysate was placed in a TRIZOL reagent to extract the total RNA. NanoDrop (Thermo Scientific, Waltham, MA, USA) and TOPscript™ RT DryMIX (dT18 plus) were used to analyze the concentration of mRNA and synthesize cDNA, respectively. Real-time PCR reactions operated in a LightCycler 480 (Roche, Basel, Switzerland) instrument using TB Green^®^ Premix Ex Taq™ II (Tli RNaseH Plus). *gapdh* was used as a housekeeping gene, and the mRNA level of each gene was normalized compared to *gapdh*. The calculation equation for gene expression is as follows: 2−ΔΔCT, where ΔΔCT = (CT*target*−CT*gapdh*) at time x−(CT*target*−CT*gapdh*) at time 0, time x represents any time point, and time 0 represents the 1 X expression of the gene in the untreated group normalized to *gapdh*. The experiment was conducted three times separately. The primers are displayed in Table S2. 

### 2.13. Western Blot Analysis

RAW 264.7 cells were pretreated with or without AD (5–40 µM) and stimulated with RANKL (50 ng/mL) to detect the activation of MAPK. The total lysate was prepared using RIPA buffer containing protease inhibitors for 30 min on ice followed by centrifugation at 14,000 rpm for 20 min at 4 °C. Then, the Bradford assay was used to measure the protein concentration according to the instruction of the manufacturer. An equal amount of protein for each sample was separated in 12% SDS-PAGE gel. After electrophoresis, separated proteins were transferred onto the PVDF membrane (Bio-Rad, Hercules, CA, USA). The membrane was blocked in 5% skim milk for 1 h at room temperature. The membranes were incubated with anti-BIP, anti-phospho-eIF2α, and anti-eIF2α to detect the expression of ER stress; anti-NFATc1 and anti-c-fos to detect osteoclast transcription factors; and anti-phospho-ERK, anti-ERK, anti-phospho-JNK, JNK, anti-phospho-p38, and anti-p38 to detect expression of MAPK. Furthermore, β-actin was used as the housekeeping gene. Then, the membranes were incubated overnight at 4^o^C before being washed in TBS-T followed by the incubation with secondary antibodies. The antibody-bounded proteins were visualized with ECL Western blotting detection reagents (Thermo Fisher Scientific, Waltham, MA, USA) using ImageQuant LAS 4000 (GE Healthcare, Chicago, IL, USA). ImageJ software was employed to quantify the observed band and normalized with control.

### 2.14. Statistics

The Sigma Plot 12.1 Student’s *t*-test was used to calculate statistical significance. The data were expressed as means ± standard deviation (SD) for at least three independent experiments, with *p* < 0.05 being considered as statistically significant.

## 3. Results

### 3.1. The Specific Interaction between AD and RANKL

**Figure 1:**
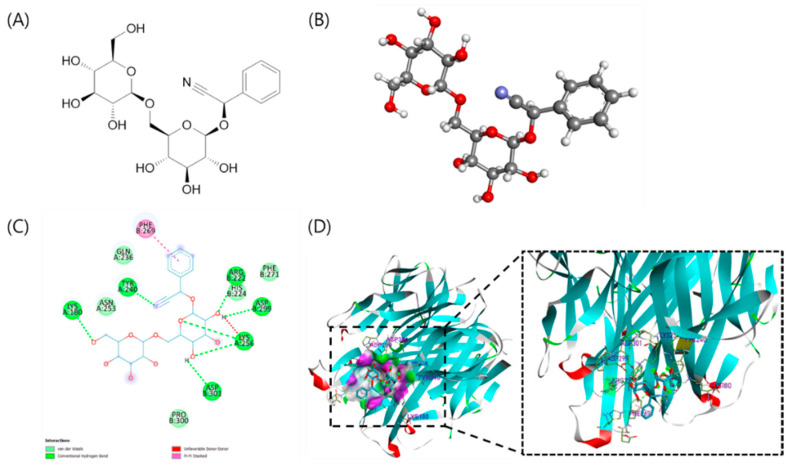
Molecular docking simulation of the interaction between AD and the 1IQA protein of RANKL expression. Structure of amygdalin (AD) (**A**) in 2D and (**B**) 3D. Interactions between AD and associated residues in the interface of the homology model for RANKL proteins in (**C**) 2D and (**D**) 3D were generated using the Discovery Studio.

Since RANKL is a key molecule that regulates the differentiation and function of osteoclast, understanding the effect of Amygdalin (AD) (Figure 1A,B) on RANKL is important for further in-depth studies. Therefore, AutoDock Vina 1.1.2 was employed to predict the binding site of AD and RANKL. Based on the basic analysis published, the virtual structure of RANKL (PDB ID: 1IQA) was suitably chosen as the receptor structure for virtual screening. Previous studies indicated that the solvent-accessible surface loops of RANKL are unlike any other TNF family member, exhibiting significantly different lengths and conformations: the AA′′ loop (residues Asn170–Ala193, bridging β-strands A and A′′), the CD loop (residues His224–Asp233), the DE loop (residues Ser245–Ser251), and the EF loop (residues Lys261–Phe269) [27]. Experiments using RANKL mutants have shown that the efficiency of RANKL was significantly decreased by a single amino acid replacement in the DE loop. Moreover, RANKL with either a deletion or a replacement in the AA′′ loop (from Gly177 to Leu183) failed to induce osteoclast precursors to differentiate in vitro [28]. Besides, three residues are important for the interaction between ligand and receptor, including Ile248 in the DE loop for conserved interaction, Lys180 in the AA′′ loop, and Gln236 in the N-terminal of D strand for specific interaction [26].

According to the docking result, the binding affinity and the RMSD between AD and RANKL were −6.4 kcal/mol and 0.771 Å, respectively. AD-established hydrogen bonds interacted with Lys180, Arg222, Tyr240, Lys256, Asp299, and Asp 301 with bond distances of 2.16, 2.53, 2.54, 2.79, 2.41, and 2.35 Å, respectively. Additionally, AD displayed van der Waals interactions with His224, Gln236, Asn253, Phe271, and Pro300. π–π interaction was known for keeping the stability of aromatic interaction [29]. The docking result indicated that AD also formed a π–π interaction with Phe269 with a bond distance of 3.67 Å (Figure 1C,D). Molecular docking simulation between AD and RANKL suggested that AD may be a potential compound for the inhibition of RANKL. 

### 3.2. The Cytotoxic Effect and Cell Confluency of AD in RAW264.7 Cells

**Figure 2:**
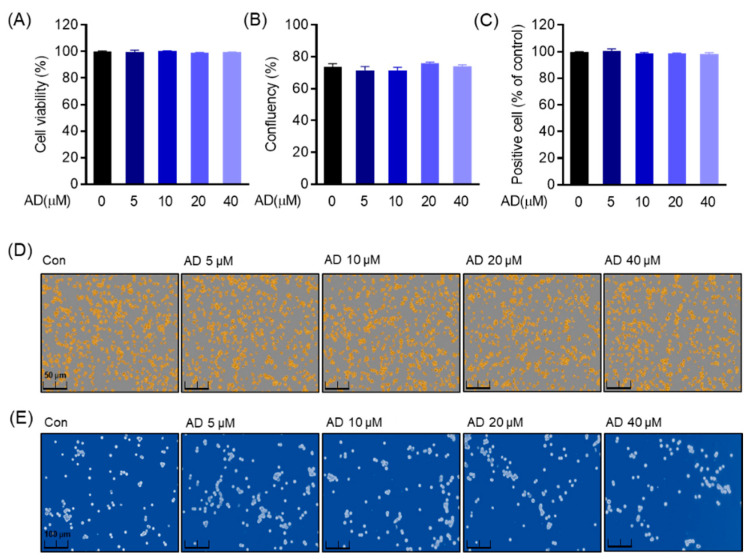
Cell viability and confluency. Cytotoxic effect of AD at the indicated concentration after treating for 5 days at the density of 1 × 10^3^ cells/well in the 96-well plate. (**A**) Measurement of cell viability by MTT. (**B**,**D**) Cell confluency measurement by Incucyte^®^ Live Cell analysis systems. (**C**,**E**) Positive cell measurement through trypan blue staining analysis.

Since cytotoxicity is unwanted property of immune modulators, before carrying out further experiments on the effect of AD on osteoclast differentiation, we first determined the cytotoxicity of AD on RAW 264.7 cells. As shown in Figure 2A–C, it was confirmed that RAW 264.7 cells were not cytotoxic at concentrations ranging from 5 μM to 40 μM through MTT analysis, cell confluency, and trypan blue staining after AD treatment for 5 days. In addition, in order to confirm the cytotoxic effect of AD on RAW 264.7 cells, it was confirmed that AD maintains stable confluency and the number of positive cells within the indicated concentrations through the Incucyte^®^ Live Cell assay system and trypan blue staining (Figure 2D,E). Therefore, we confirm that AD exerted no toxicity on RAW 264.7 at this indicated experimental concentration.

### 3.3. Inhibitory Effect of AD on RANKL-Induced Osteoclast Differentiation and Formation

**Figure 3:**
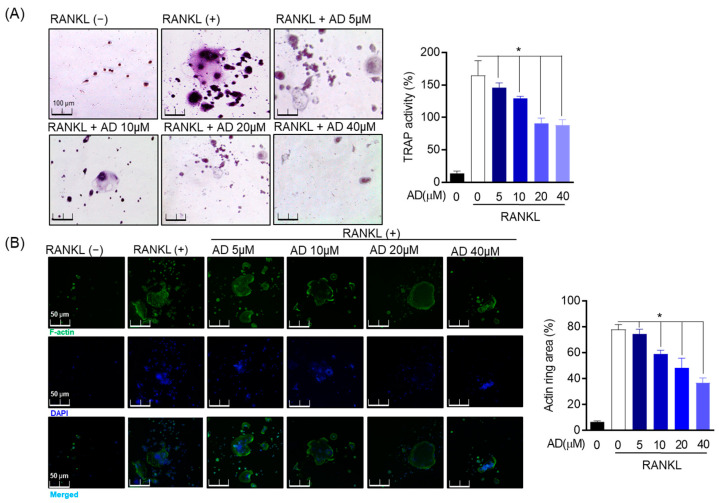
Inhibitory effect of AD on RANKL-induced osteoclast differentiation and formation. (**A**) RAW 264.7 cells (1 × 10^3^ cells/well) were cultured in 24-well culture plates, then treated with AD (5–40 µM) for 5 days. After culture, TRAP staining and activity were analyzed. (**B**) RAW 264.7 cells were treated in the presence or absence of AD and stimulation of RANKL (50 ng/mL) for 5 days to investigate the formation of osteoclast, Alexa 488-Phalloidin (green), and DAPI (blue) was used to stain the actin cytoskeleton and counterstained the nuclei, respectively. * *p* < 0.05, versus the RANKL-treated group.

To ascertain whether AD is involved in the process of osteoclastogenesis, we created an osteoclastogenesis model using RANKL-induced osteoclast. Tartrate resistant acid phosphatase (TRAP) is considered as a chemical marker for osteoclast differentiation and is used as an enzyme marker to evaluate osteoclast differentiation. As shown in Figure 3A, RAW 264.7 cells commenced becoming TRAP-positive mature multinucleated giant cells in the positive control group. TRAP generation and activity were suppressed in the treatment with AD in a concentration-dependent manner. To evaluate the osteoclast-specific structure, structural dynamics of the actin cytoskeleton in mature osteoclasts were assessed by fluorescence staining by Alexa 488 Phalloidin. Besides, DAPI was used to stain the nucleus of mature osteoclast. The data revealed that RANKL caused mature osteoclast surrounded by the inactive podosome actin belt to be impeded by RANKL in a concentration-dependent manner. To be specific, a large F-actin ring of RAW 264.7 was detected following RANKL stimulation in the control group while the area of actin ring pits was diminished by AD intervention. Furthermore, DAPI staining exhibited a similar result (Figure 3B). Therefore, our data confirm that AD has an inhibitory effect on the differentiation and formation process of the RANKL-induced osteoclast.

### 3.4. Inhibitory Effect of AD on the Function of Osteoclast in RAW 264.7

**Figure 4:**
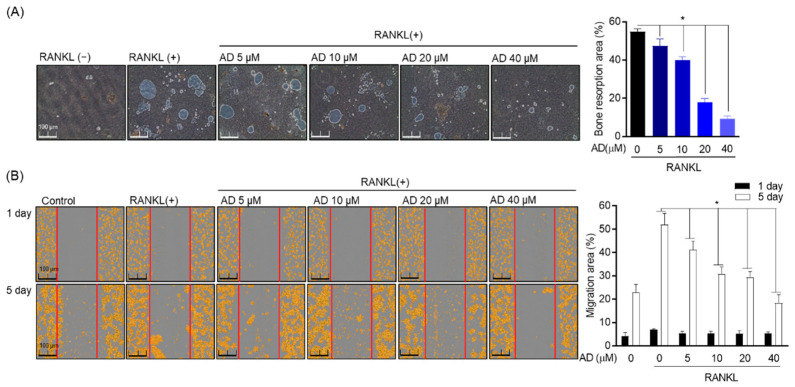
Inhibitory effect of AD on RANKL-induced osteoclast of functions. (**A)** RAW 264.7 (1 × 10^3^ cells/well) were cultured in osteo-surface well and treat with RANKL (50 ng/mL) to activate osteoclast formation and incubate for 7 days. Incucyte^®^ Live Cell analysis systems and ImageJ program were used to observe and quantify the percentage of bone resorption pit area to the total osteo-surface well area. (**B**) Red lines indicate the migration borders at the beginning of the assay. Cell migration was analyzed on the first day and the fifth day after treatment. * *p* < 0.05, versus the RANKL-treated group.

Next, the function of osteoclast was investigated by culturing RAW 264.7 cells in osteo surface well treated with RANKL (50 ng/mL) to activate osteoclast formation and incubated for 7 days. Then, the resorption area was visualized and evaluated using the microscope and ImageJ software. The result showed that the resorption area was remarkably reduced in the indicated concentration of AD-treated cells (Figure 4A). Besides, the cell migration assay was used to evaluate the action of osteoclast. Cell migration was determined by Incucyte^®^ Live Cell analysis systems and evaluated by ImageJ software on the first day and the fifth day after treatment. The migration of osteoclast in RAW 264.7 cells was gradually decreased at the higher concentration of AD. Compared to the first day after treatment, cell migration on the fifth day showed a higher density of cells without migration, suggesting that AD suppresses the function of osteoclast in a concentration-dependent manner (Figure 4B).

### 3.5. Inhibitory Effect of Amygdalin on ER Stress in RANKL-Induced Osteoclast

**Figure 5:**
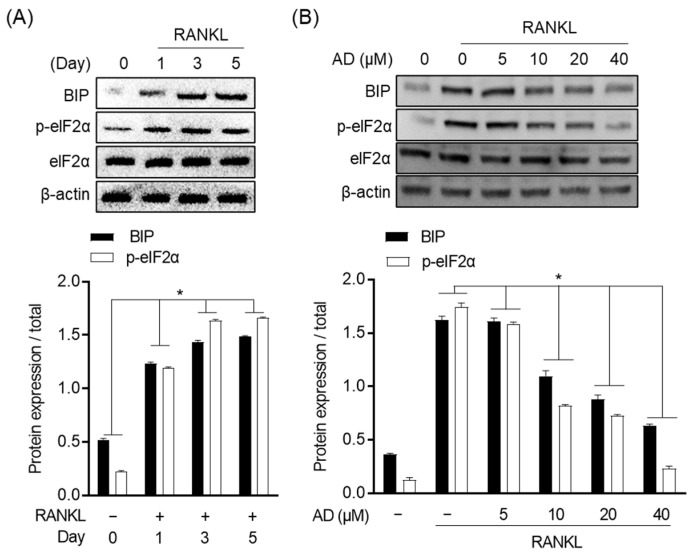
Inhibitory effect of AD on ER stress in RANKL-induced osteoclast. RAW 264.7 cells (1 × 10^4^ cell/mL) were seeded in 6-well plate and incubated for 24 h. (**A**) Cells were treated with RANKL (50 ng/mL) for 1, 3, and 5 days. (**B**) Cells were incubated with the indicated AD concentration with the stimulation of RANKL (50 ng/mL) for 5 days. Cells then were subjected to Western blot analysis to quantify the expression, and the relative of protein expression was measured by ImageJ software. * *p* < 0.05, versus the RANKL-treated group.

ER stress plays a vital role in regulating osteoclast differentiation. The activation of ER stress promotes osteoclast differentiation through the activation of immunoglobin heavy-chain-binding protein (BIP) and α-subunit of eukaryotic initiation factor 2 (eIF2α). In this study, Western blot was used to assess the inhibitory effect of AD on ER stress in RANKL-induced osteoclast through the expression of BIP, p-eIF2α, and eIF2α. As shown in Figure 5A, the expression of BIP and the phosphorylation of eIF2α were upregulated by RANKL stimulation while the protein expression of both BIP and p-eIF2α was decreased in the treatment with AD at a concentration-dependent manner (Figure 5B). These data suggested that AD ameliorates ER stress in RANKL-induced osteoclast. 

### 3.6. Inhibitory Effect of AD on Oxidative Stress Markers in RANKL-Induced Osteoclast

**Figure 6:**
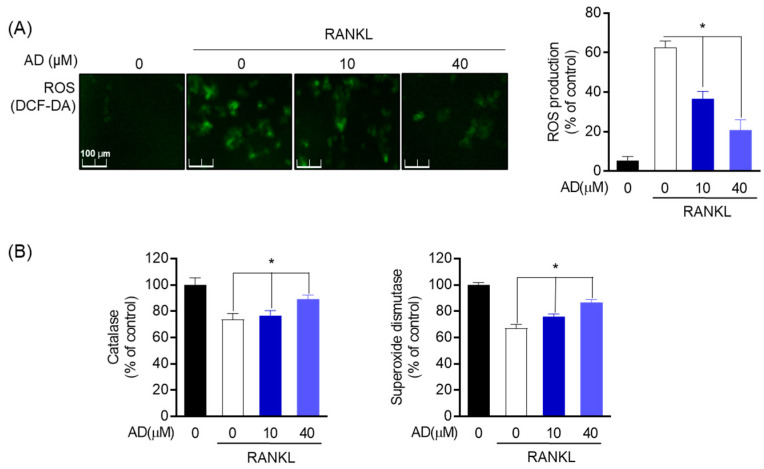
Inhibitory effect of AD on oxidative stress markers in RANKL-induced osteoclasts. (**A**) DCF-DA staining was utilized to detect the RANKL-induced ROS generation in RAW264.7 with different indicated concentrations of AD. (**B**) SOD and CAT activity levels of RAW 264.7 cells pre-treated with AD and stimulated by RANKL (50 ng/mL). * *p* < 0.05, versus the RANKL-treated group.

Reactive oxygen species (ROS) are not only the necessary intracellular secondary messengers but also important components that promote the differentiation of osteoclast. In this study, DCF-DA was used to evaluate the generation of ROS in RANKL-stimulated cells as well as the effect on antioxidant enzyme-related proteins and genes. ROS accumulation was increased in the presence of RANKL; however, in the treatment with AD, it was decreased (Figure 6A). Hence, to assert the effect of AD on oxidative stress, antioxidant enzymes, including catalase (CAT) and superoxide dismutase (SOD), were employed. CAT is included in decaying intracellular hydrogen peroxide and conserving normal ROS levels to lessen toxicity reaction while the activity of SOD mirrors the ability to scavenge oxygen free radicals. As shown in Figure 6B, SOD and CAT were gradually increased in the treatment with AD at a dose-dependent manner which means that AD stimulated the gene expression of SOD and CAT. Interestingly, at the concentration of 40 µM, SOD showed a notable up-regulation compared to the RANKL-treated group. Therefore, these results verify that AD is not only involved in the process of oxidative stress but also suppresses oxidative markers in RANKL-induced osteoclast. 

### 3.7. AD Downregulates the Expression of Osteoclast Marker Genes and Transcript Factors

**Figure 7:**
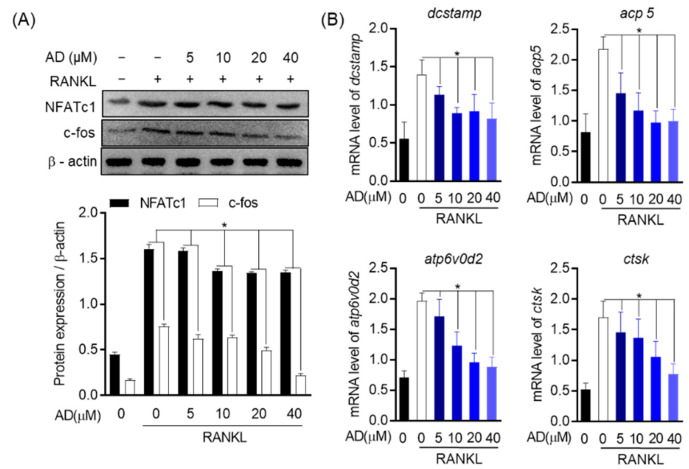
AD decreases the expression of osteoclast-specific genes and transcription factors. (**A**) Total proteins from RAW264.7 stimulated with or without RANKL or with indicated AD concentration for 24 h. The untreated with neither AD nor RANKL group was used as the negative control. Cell lysates were then analyzed by Western blot with the indicated antibodies. The protein expression of c-Fos and NFATc1 relative to β -actin, a housekeeping gene, was quantified by densitometry. (**B**) The mRNA level of osteoclast-specific genes, including *dcstamp*, *acp5*, *ATP6v0d2*, and *ctsk*, was analyzed by real-time PCR. * *p* < 0.05 versus the RANKL-treated group.

To elucidate whether AD downregulates the expression of osteoclast markers gene and transcription factor, the effect of AD on RANKL-induced osteoclast-specific transcription factors and osteoclast-specific genes were investigated. Nuclear factor of activated T cells 1 (NFATc1) and c-fos are particular and required transcription factors for osteoclast formation as well as osteoclastogenesis. Therefore, in this study, the effect of AD on osteoclast-specific marker genes and transcription factors was investigated. As shown in Figure 7A, β-actin was used as an internal control for cytosolic fraction, and the expression of NFATc1 and c-fos was dose-dependently downregulated by the treatment of AD. Notably, increased c-fos expression in RANKL-induced osteoclast was remarkably inhibited by the treatment of 40 µM AD. In addition, real-time PCR was used to analyze the expression of osteoclast-specific genes, such as dendritic cell-specific transmembrane protein (*dcstamp*), acid phosphatase 5 (*acp5*), ATPase H+ Transporting V0 Subunit D2 (*ATP6v0d2*), and cathepsin K (*ctsk*). The data indicated that the level of osteoclast-specific genes was reduced in a concentration-dependent manner (Figure 7B). Consequently, these results confirm that, in the process of osteoclast formation, AD impedes osteoclast formation, leading to its potential in terms of osteoclastogenesis treatment.

### 3.8. Suppression of AD on RANKL-Induced MAPK Activation

**Figure 8:**
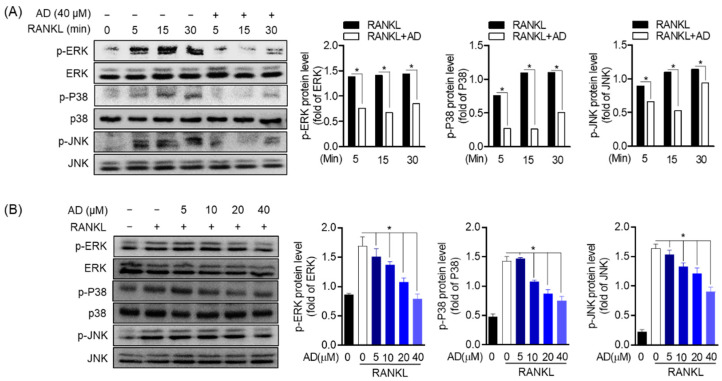
AD decreases the expression of RANKL-induced MAPKs activation. RAW 264.7 cells (5 × 10^5^ cell/mL) were seeded in 6-well plate and incubated for 24 h. (**A**) Cells were pre-incubated with AD (40 µM) for 2 h before treating with or without RANKL (50 ng/mL) for 5, 15, and 30 min. (**B**) Cells were treated in the presence or absence of AD at the indicated concentration, then stimulated with or without RANKL (50 ng/mL). Then, cell lysates were subjected to Western blot analyses to detect the activation of MAPK. * *p* < 0.05 versus the RANKL-treated group.

Mitogen-activated protein kinase (MAPK), the old cell signaling pathway, is involved in the process of osteoclast differentiation, function, and osteoclastogenesis. There are three main types of MAPK, including extracellular signal-regulated kinase (ERK), c-Jun N-terminal kinase (JNK), and p38. The activation of MAPK in response to ER stress. Therefore, to ascertain the involvement of AD in MAPK, two sets of experiments were conducted, including in a time-dependent and concentration-dependent manner. As shown in Figure 8A, in a time-dependent manner, the protein expression of p-ERK, p-p38, and p-JNK was upregulated in the presence of RANKL (50 ng/mL), while in the treatment with AD, the expression is significantly decreased. In a concentration-dependent manner, the data showed that AD inhibited the phosphorylation of the MAPK signaling pathway compared to the total form (Figure 8B). Therefore, these results affirm that AD is not only involved in, but also has a potent suppressive effect on, the MAPK signaling pathway, which in turn leads to its prospective natural-derived treatment of osteoclast bone-related diseases.

## 4. Discussion

Under physiological conditions, through a balance between bone formation by osteoblast and bone resorption by osteoclast, the maintenance of bone mass is achieved [30]. Osteoclast, the principle of bone resorption in the body, plays an important role in skeletal development and maintenance regulates osteoblast differentiation, promotes the mobilization of hematopoietic stem cell from the bone marrow to the bloodstream, and is involved in the response of the immune system. In some cases, this balance is disrupted, and the activity of osteoclast is over-expressed or abnormal, leading to the activation of numerous bone metabolic disorders and localized bone diseases [30,31,32,33]. Thus, understanding the mechanism underlying osteoclast and bone-related disorder is crucial for the development of natural products as a treatment for osteoclastogenesis and bone diseases.

In this study, since the in silico study of AD and osteoclast is currently unknown, the computational screening of AD and its properties related to osteoclast differentiation was first assessed. The AA” loop in protein RANKL was reported to have an important role in osteoclast differentiation [26,29]. By molecular docking, the result predicted that AD effectively binds to the active site of RANKL, which has been known as the key regulator of osteoclast precursors. Additionally, in 2021, Abbas et al. reported that resveratrol, a common secondary metabolite found in red grapes, red wine, and peanuts, was exhibited as a potential compound for drug preparation against bone loss by using in vitro and in silico methods. By using molecular docking simulation between resveratrol and eight RANKL receptors (PDB ID: 1JTZ, 1S55, 3ME2, 3QBQ, 4E4D, 4GIQ, 1IQA, and 1TJZ), resveratrol showed the high binding affinities from −7.6 to −6.7 kcal/mol [34]. Besides, in 2019, El-Baz et al. revealed that astaxanthin-rich fraction from *Heamatococcus pluvialis* shows benefits in the control of age-related osteoporosis, not only through preserving bone mass and serum calcium/phosphorus level but also through an increase in the bone mineralization rate. Especially, all-trans astaxanthin, the major carotenoid found in *H. pluvialis*, possessed a high binding affinity towards the OPG-RANKL complex protein with the free energy of binding −6.3 kcal/mol [35]. Our study revealed that AD showed high binding affinities with 1IQA, the crystal structure of the extracellular domain of mouse RANK ligand, of −6.4 kcal/mol. These results suggested that AD may be a potential compound for the inhibition of RANKL. 

Then, the in vitro experiment on RAW264.7 cell model was carried out to study the effect of AD on osteoclast differentiation. RANKL, a central positive regulator of osteoclast produced by osteoblast-lineage cells, binds to its receptor RANK on the surface of myeloid cells, stimulating their differentiation into osteoclasts which results in the activation of osteoclast formation along with the rapid recruitment of multiple intracellular signaling molecules [32,33,36,37]. TRAP is the mature osteoclast marker [30]. The data revealed that RANKL-induced cells were able to form TRAP-positive osteoclast and that this process was inhibited by AD at this indicated experimental concentration. Osteoclast differentiation is regulated by various upstream signaling of MAPK, including JNK, ERK, and p38 [38]. RANKL-induced bone resorption is critically regulated through MAPK induced c-Fos expression [36]. NFATc1 is the crucial downstream signaling event in RANKL-mediated ROS signaling, and its expression is regulated by c-Fos/c-Jun. Thus, increased NFATc1 induces the formation of TRAP-positive osteoclast cells [36]. In this study, AD suppresses the phosphorylation of MAPK activation and osteoclast differentiate downstream signal, including c-Fos and NFATc1. RANKL-induced osteoclast differentiation through oxidative stress and ER stress in the regulation of osteoclast differentiation is reported, relating to the inflammatory process [36,39]. ROS is known as a pivotal regulator of ER function along with ER stress, and increased ROS production occurs concurrently [6,40]. The results suggested that the inhibitory effect of AD was exhibited by suppressed ROS accumulation by DCF-DA staining and the activation of antioxidant enzymes, such as CAT and SOD. In addition, in the presence of AD, the expression of cytosolic protein, including BIP and eIF2α, was significantly inhibited.

In conclusion, these data demonstrated that AD inhibited osteoclast by RANKL-induced osteoclast in the RAW264.7 cell model. The treatment of AD not only suppresses the structure and function of osteoclast but also the expression osteoclast-specific genes and transcription factors, thereby positively influencing the treatment of osteoporosis for further study. Additionally, the generation of ROS and ER stress was strongly inhibited by AD. Finally, AD downregulates the activation of MAPK which is an old cell signaling pathway related to osteoclastogenesis. Our study suggests that amygdalin (AD), a secondary compound isolated from seeds of *P. armeniaca* L., has the potential to become a natural-derived treatment for osteoclastogenesis and bone disorder diseases.

## Data Availability

Not applicable.

## Supplementary Materials

The following supporting information can be downloaded at: https://www.mdpi.com/article/10.3390/biom12020256/s1, Figure S1: ^1^H NMR spectrum of AD (500MHz, DMSO-*d*_6_); Figure S2: ^13^C NMR spectrum of AD (500MHz, DMSO-*d*_6_); Table S1: ^1^H (500 MHz) and ^13^C-NMR (500 MHz) NMR data of amygdalin in DMSO-*d*_6_; Table S2: Primer sequence of real-time quantitative PCR analysis.
