# Supplementary material for: Effect on Osteoclast Differentiation and ER Stress Downregulation by Amygdalin and RANKL Binding Interaction
Source: Biomolecules. 2022 Feb 4;12(2):256. doi: 10.3390/biom12020256 (PMC8961616; doi:10.3390/biom12020256)
Supplement: Supplementary file 1 [file biomolecules-12-00256-s001.zip › biomolecules-1570589-supplementary.pdf]

Supplementary file

Effect of osteoclast differentiation and ER stress downregulation by amygdalin and RANKL binding interaction

Nguyen Minh Trang [1†], Eun-Nam Kim [1†], Hyun-Su Lee [2] and Gil-Saeng Jeong [3,*]

[1] *College of Pharmacy, Keimyung University, Daegu 42601, Korea; ngminhtrang52@gmail.com (N.M.T.); enkimpharm@gmail.com (E.-N.K.)*

[2] *School of Medicine, Kyungpook National University, Daegu 41566, Korea; hyunsu.lee@knu.ac.kr (H.-S.L.)*

[3] *College of Pharmacy, Chungnam National University, Daejeon 34134, Korea; gsjeong@cnu.ac.kr (G.-S.J.)*

***** Correspondence: gsjeong@cnu.ac.kr (G.-S.J.)

† These authors contributed equally to this work.

List of Figures and Tables

Figure S1. ^{1}H NMR spectrum of AD (500MHz, DMSO-d_6)

Figure S2. ^{13}C NMR spectrum of AD (500MHz, DMSO-d_6)

Table S1. ^{1}H (500 MHz) and ^{13}C-NMR (500 MHz) NMR data of amygdalin in DMSO-d_6.

Table S2. Primer sequence of real-time quantitative PCR analysis.

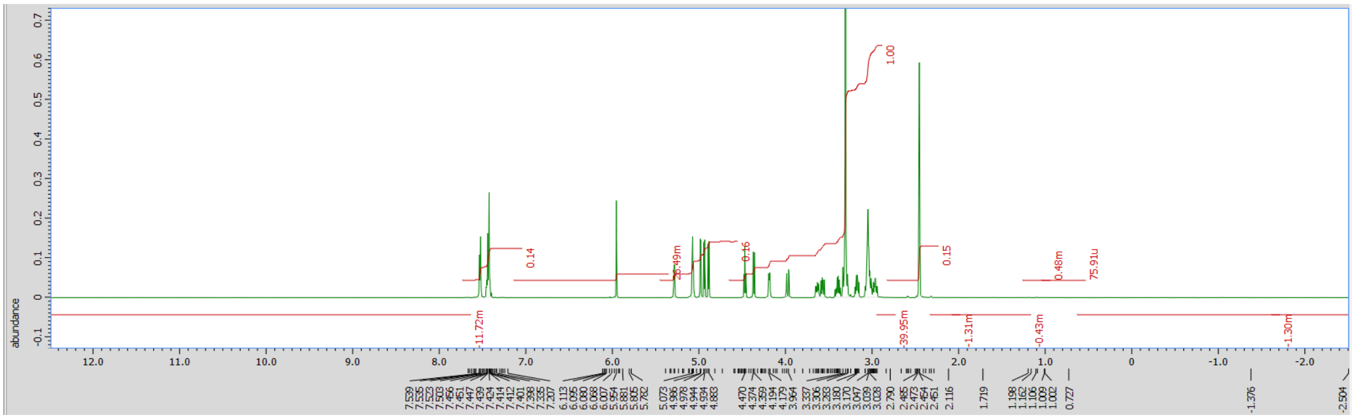

Figure S1. ^{1}H NMR spectrum of AD (500MHz, DMSO-d_6).

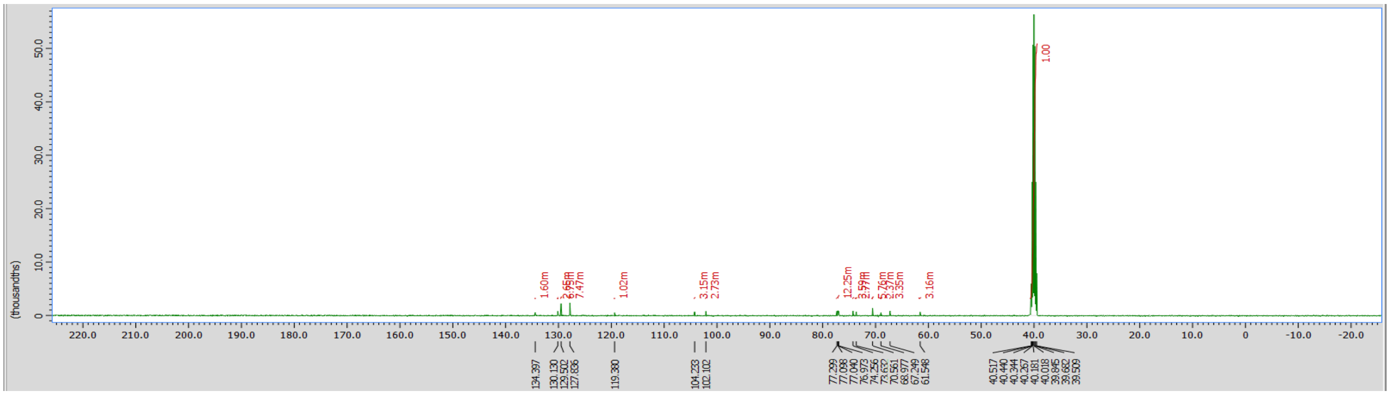

Figure S2. ^{13}C NMR spectrum of AD (500MHz, DMSO-d_6).

Table S1. ^{1}H (500 MHz) and ^{13}C-NMR (500 MHz) NMR data of amygdalin in DMSO-*d6*.

Position	Amygdalin	
	**δ_C**	**δ_H (J in Hz)**
1	134.40	6.00 (1H, s)
2	127.84[b]	7.54 (1H, m)[d]
3	129.50[a]	7.42 (2H, m)
4	130.13	7.44 (3H, m)[c]
5	129.50[a]	7.44 (3H, m)[c]
6	127.84[b]	7.54 (3H, m)[d]
7	68.98	7.45 (2H, m)
8	119.38	–
1′	102.10	4.47 (1H, d, 7.9)
2′	73.63	3.37 (1H, m)
3′	76.97	3.46 (1H, m)
4′	70.10	3.52 (1H, m)
5′	76.97	3.59 (1H, m)
6′	69.00	4.19 (1H, d, 10.7) / 3.96 (1H, m)
1″	104.23	4.37 (1H, d, 8.1)
2″	74.26	3.30 (1H, m)
3″	77.10	3.55 (1H, m)
4″	70.56	3.43 (1H, m)
5″	77.10	3.49 (1H, m)
6″	61.55	4.17 (1H, m) / 3.74 (1H, dd, 12.2, 5.2)

[a,b,c,d] Overlapped signals

Table S2. Primer sequences of real-time quantitative PCR analysis.

Target Gene		Sequence (5′-3′)
dcstamp	Forward	TTTGCCGCTGTGGACTATCTGC
	Reverse	GCAGAATCATGGACGACTCCTTG
acp5	Forward	CGTCTCTGCACAGATTGCAT
	Reverse	GAGTTGCCACACAGCATCAC
atp6v0d2	Forward	TGTGTCCCATTCTTGAGTTTGAGG
	Reverse	AGG GTCTCCCTGTCTTCTTTGCTT
ctsk	Forward	TCCGCAATCCTTACCGAATA
	Reverse	AACTTGAACACCCACATCCTG
gapdh	Forward	ACAGTCCATGCCATCACTGCC
	Reverse	GCCTGCTTCACCACCTTCTTG
